# Annealing Effects of Parylene-Caulked Polydimethylsiloxane as a Substrate of Electrodes

**DOI:** 10.3390/s16122181

**Published:** 2016-12-18

**Authors:** Jinmo Jeong, Namsun Chou, Gihyun Lee, Sohee Kim

**Affiliations:** 1 School of Mechanical Engineering, Gwangju Institute of Science and Technology (GIST), Gwangju 61005, Korea; jeongjm@gist.ac.kr; 2 Department of Robotics Engineering, Daegu Gyeongbuk Institute of Science and Technology (DGIST), Daegu 42988, Korea; namsunchou@gmail.com; 3 Department of Biomedical Science and Engineering, Gwangju Institute of Science and Technology (GIST), Gwangju 61005, Korea; leegihyun@gist.ac.kr

**Keywords:** implantable electrode, polydimethylsiloxane (PDMS), parylene, parylene-caulked PDMS (pc-PDMS), annealing, mechanical strength, electrochemical impedance spectroscopy (EIS), stability

## Abstract

This paper investigates the effects of annealing of the electrodes based on parylene-caulked polydimethylsiloxane (pc-PDMS) in terms of mechanical strength and long-term electrical property. Previously, the electrodes based on pc-PDMS showed a better ability to withstand in vivo environments because of the low water absorption and beneficial mechanical properties of the substrate, compared to native PDMS. Moreover, annealing is expected to even strengthen the mechanical strength and lower the water absorption of the pc-PDMS substrate. To characterize the mechanical strength and water absorption of the annealed pc-PDMS, tensile tests were carried out and infrared (IR) spectra were measured using Fourier transform infrared spectroscopy over a month. The results showed that annealed pc-PDMS had higher mechanical strength and lower water absorption than non-annealed pc-PDMS. Then, electrochemical impedance spectroscopy was measured to evaluate the electrical stability of the electrodes based on annealed pc-PDMS in phosphate-buffered saline solution at 36.5 °C. The impedance magnitude of the electrodes on annealed pc-PDMS was twice higher than that of the electrodes on non-annealed pc-PDMS in the initial days, but the impedance magnitude of the electrodes based on two different substrates converged to a similar value after eight months, indicating that the annealing effects disappear after a certain period of time in a physiological environment.

## 1. Introduction

In neural signal recording using implantable electrodes based on polymers, the durability and low water absorption of the electrode substrate have a major impact on the recording performance. To endure the in vivo environment where peristalsis of internal organs and muscles exists, high tensile strength and adhesion between electrodes and the substrate are desired [1,2]. The water absorption into the substrate of the electrodes has interrupted the biomedical signal recording performance for long-term in vivo use. The impedance magnitude of the electrodes may decrease when used in an in vivo environment for a long time because of water absorption [3,4]. The impedance magnitude of the electrodes also has a major impact on bio-signal recording. A low impedance magnitude leads to high sensitivity while a high impedance magnitude leads to high selectivity [5,6]. In general, a relatively high level of electrode impedance is required when they are used for recording bio-signals in a focused region. Further, the high selectivity allows for recording signals from a small population of neurons without activating neighboring populations [6]. The reduction of the electrode active area could be a solution to increase the impedance magnitude. In addition, annealing could also produce a higher impedance of the electrodes [7,8]. The impedance magnitude could also be a major factor for precision sensors such as humidity sensors to measure the signal in a noisy environment, and using the optimized circuit could be a solution to obtaining precise results [9,10]. Annealing has been commonly used to optimize polymers because it not only increases the mechanical strength and adhesion strength between the metal and polymer, which leads to good durability of the electrodes based on a polymer, but it increases the impedance magnitude [11,12].

Parylene-caulked polydimethylsiloxane (pc-PDMS) has been recently proposed and used as a substrate for flexible electrodes [13,14,15]. As PDMS has a porous surface structure, it exhibits properties such as high gas permeability and high water absorption [14,16,17,18,19]. Pc-PDMS can be formed as the particles of parylene in a gaseous state are trapped only in the porous structure of the PDMS surface after etching the majority of the parylene deposited conformally on the PDMS surface [17]. Pc-PDMS is proved to preserve the inherent beneficial mechanical properties of PDMS, such as elasticity and flexibility, and to lower the water absorption better than native PDMS [15]. The latter is due to the fact that parylene has a lower water absorption than native PDMS, being suitable as a substrate for implantable electrodes [13,14,15,17]. However, water absorption into pc-PDMS is also inevitable as the electrodes are based on polymeric materials, accompanying the decline in durability and impedance magnitude. As parylene trapped in the pc-PDMS is a semi-crystallized polymer, annealing could optimize the parylene by increasing the level of crystallinity. In fact, the annealing of parylene has been used to improve the mechanical strength and adhesion strength between two parylene layers [20,21]. Also, annealing metals does not only provide mechanical stability, but also high impedance, which could be beneficial in cases when relatively high impedance electrodes are necessary.

In this paper, we investigate the effects of annealing of the electrodes based on pc-PDMS. The electrodes based on annealed pc-PDMS are expected to have beneficial inherent properties of pc-PDMS with improved durability and low water absorption in comparison with pc-PDMS that is not annealed. To investigate the annealing effects in terms of the mechanical strength and water absorption of the substrate, tensile tests and Fourier transform infrared spectroscopy (FTIR) measurements were carried out. To evaluate the impedance characteristics, the electrochemical impedance spectroscopy (EIS) was measured for the electrodes based on annealed and non-annealed pc-PDMS in a physiological environment for eight months.

## 2. Materials and Methods

### 2.1. Preparation of pc-PDMS Substrate

The pc-PDMS substrate was formed by depositing parylene dimer onto the porous surface of PDMS, thereby lowering the water permeability of the substrate and maintaining the inherent beneficial properties of PDMS such as flexibility and elasticity. To produce the annealed pc-PDMS, PDMS with a ratio of 1:10 of PDMS to Sylgard 184^®^ (Dow Corning, Midland, MI, USA) was spun on a silicon wafer using a spin-coater (ACE-200, DONG AH, Seoul, Korea), and cured in a dry oven (HB-501SPS, HANBAEK, Bucheon, Korea) for 1 h at 150 °C, resulting in a thickness of 150 μm. Next, parylene in a thickness of 400 nm was deposited on the PDMS substrate using low-pressure chemical vapor deposition (LPCVD) (PDS 2010, Specialty Coating Systems, Indianapolis, IN, USA). Then, parylene on PDMS was etched using O_2_ plasma 50 W for 5 min so that the majority of parylene on the PDMS surface was removed and only a small portion of parylene remained in the porous structures of PDMS [15,17]. Lastly, after separation of pc-PDMS from the silicon wafer, it was annealed for 2 h at 200 °C in a vacuum oven (OV-11, JEIOTECH, Seoul, Korea).

### 2.2. Tensile Test

The tensile tests were carried out according to the ASTM standard [22]. Dumbbell-shaped, 2-mm-thick specimens based on annealed and non-annealed pc-PDMS were prepared, in dimensions of 115 mm × 25 mm. Eight specimens per substrate were tested using a universal tension test machine (Instron5567, Instron, Norwood, MA, USA). One end of the samples was fixed and the other end was pulled at a pulling speed of 500 mm/min until fracture of the samples. The stress was measured while the strain was increased. 

### 2.3. Fourier Transform Infrared Spectroscopy (FTIR)

FTIR spectroscopy measurements were carried out for quantitative analysis of water absorption in annealed pc-PDMS using the attenuated total reflectance (ATR) mode. Eight specimens of annealed pc-PDMS were prepared on glass slides. The samples were soaked in phosphate buffered saline (PBS) solution at 36.5 °C for about a month and their FTIR (Varian 640, Varian, Palo Alto, CA, USA) was measured periodically after removing water from the surface of the samples using a clean wipe. The FTIR measured the samples with 18 scans per spectrum and all spectra were collected using a detector by calculating the average values of 18 scans at a resolution of 4 cm^−1^ [4,23,24] and the scan range was from 4000 to 600 cm^−1^ with scan depth of about 1~2 μm [25]. Because the depth of FTIR scan was limited only to 1~2 μm, the increase of water absorbed into specimens was not shown after a month. Hence, we measured the FTIR periodically for one month.

### 2.4. Electrode Fabrication Based on pc-PDMS

**Figure 1:**
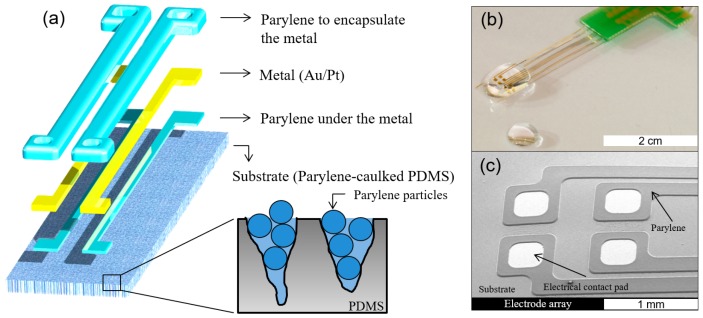
(**a**) Schematic image of the electrode array based on parylene-caulked PDMS; (**b**) photographic image of the completed electrode array connected with a printed circuit board (PCB), shown with a water droplet; and (**c**) SEM image of the completed electrodes.

The fabrication process of the electrodes based on pc-PDMS has been previously described elsewhere [13]. The electrode arrays were designed in a way that parylene encapsulated the metal traces fully except the opening areas while pc-PDMS was found at the front and back sides of the substrate except parylene-encapsulated metal traces. The thickness of the substrate was 150 μm. The parylene layer between the substrate and metal was formed using a photoresist mask against reactive ion etching (RIE) process, in a thickness of 1 μm. Pt, which shows good adhesion with parylene [13,26], and Au metal patterns were formed on the parylene-deposited substrate in thicknesses of 30 nm and 200 nm, respectively, using a lift-off process. Next, parylene layer in a thckness of 6 μm was deposited for encapsulation of the metal. After patterning the photoresist (AZ9260, AZ Electronic Materials, Darmstadt, Germany) in a thickness of about 7 μm as the mask, the electrode sites and connection pads were opened by RIE using O_2_ plasma at 50 W for 60 min, which condition was sufficient to etch the parylene away from the PDMS surface, turning the substrate into pc-PDMS at the same time. After all the fabrication processes, the electrode arrays were detached from the silicon wafer using tweezers. To form pc-PDMS on the bottom side of the electrode array, the front side of the detached electrode array was attached on a silicon wafer using sticky photoresist (AZ9260), and parylene in a thickness of 400 nm was again deposited and removed using RIE process, as described previously. Lastly, annealing was performed for 2 h at 200 °C in a vacuum oven. After annealing, the thickness of parylene encapsulating the metal patterns reduced to about 4.5 μm from 6 μm, as reported in a previous study [27]. The schematic and photographic images of the electrode array are shown in Figure 1.

### 2.5. Impedance Characterization

Electrochemical impedance spectroscopy (EIS) was performed by using an electrochemical impedance measurement system (Reference 600, Gamry, Warminster, PA, USA) to monitor the long-term electrical stability of the electrodes based on annealed pc-PDMS in 0.9% phosphate buffered saline (PBS) solution at 36.5 °C. To prevent the evaporation of solution over time, the electrodes were housed in a polypropylene container with a custom-made Teflon cap. Using the three-electrode setup, a voltage of 40 mV_rms_ was applied in the range of frequency sweep from 100 kHz to 1 Hz. Three electrodes selected from two electrode arrays were measured per substrate. And, the impedance changes were monitored at 1 Hz, which are within the frequency range of electrocardiogram (ECG) and electroencephalogram (EEG), and at 1 kHz that is within the range of internal electromyogram [28,29].

## 3. Results

### 3.1. Mechanical Characterization

**Figure 2:**
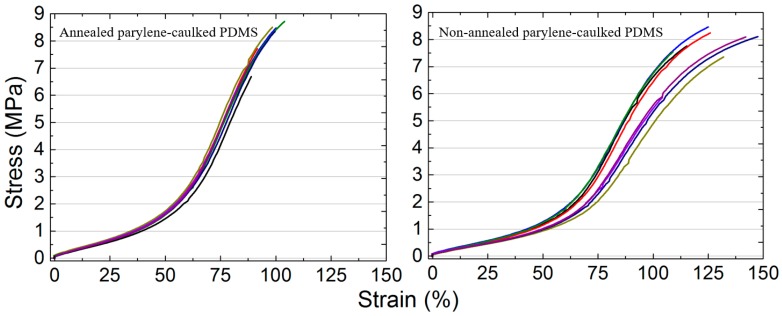
Strain-stress curves of annealed (**Left**) and non-annealed parylene-caulked PDMS (**Right**). The eight lines in different colors represent the strain-stress curves of eight different specimens.

The tensile test results are shown in Figure 2. The mechanical properties of native PDMS were reported in a previous study [15], where the maximum stress and strain were 7.65 MPa and about 125%, respectively. These values were similar to those of pc-PDMS [15]. The strain-stress curves of annealed pc-PDMS exhibited smaller variations among measurements than non-annealed pc-PDMS. Annealed and non-annealed pc-PDMS ruptured at stresses of 7.95 ± 0.77 MPa and 7.62 ± 0.88 MPa, respectively, indicating that the maximum stresses of annealed pc-PDMS were only slightly higher than that of pc-PDMS. The maximum strains of annealed and non-annealed pc-PDMS were 95.85% ± 6.10% and 121.90% ± 13.26%, respectively, indicating that annealing decreased the stretchability. The Young’s moduli of annealed and non-annealed pc-PDMS were calculated to be 2.84 ± 0.13 MPa and 1.93 ± 0.21 MPa, respectively, in the linear region of the strain-stress curves [30,31] up to strain level of 50%. It indicates that the mechanical strength of annealed pc-PDMS was higher than that of non-annealed pc-PDMS, by 47%. Based on these results, the annealing process increased the mechanical strength of pc-PDMS until 50% of the strain, which is reported to be the typical strain of polymer-based implantable devices [32,33,34]. Although the stretchability of annealed pc-PDMS was lower than that of non-annealed pc-PDMS, the stretchability of annealed pc-PDMS was about 96%, still higher than the typical values mentioned for implantable devices, and could be properly used for implantable devices where stretchability is required.

### 3.2. Water Absorption Property

Pc-PDMS consists of PDMS and parylene on its surface [3]. PDMS can be identified by functional groups such as methyl (CH_3_) and siloxane, which are represented by rocking, deformation, and asymmetric stretching of CH_3_ at wavenumbers 789–796 cm^−1^, 1260–1259 cm^−1^, and 2950–2960 cm^−1^ and Si–O–Si stretching at wavenumber 1020–1074 cm^−1^ [35]. Parylene consists of CH_2_ and benzene represented by stretching and deformation of C–H at wavenumbers 2850–3100 cm^−1^ and 1344 cm^−1^, C deformation at 1404 cm^−1^, benzene breathing at 964 cm^−1^, C–C stretching at 1480–1510 cm^−1^, and C–H_2_ bending at 1480–1510 cm^−1^ [21].

**Figure 3:**
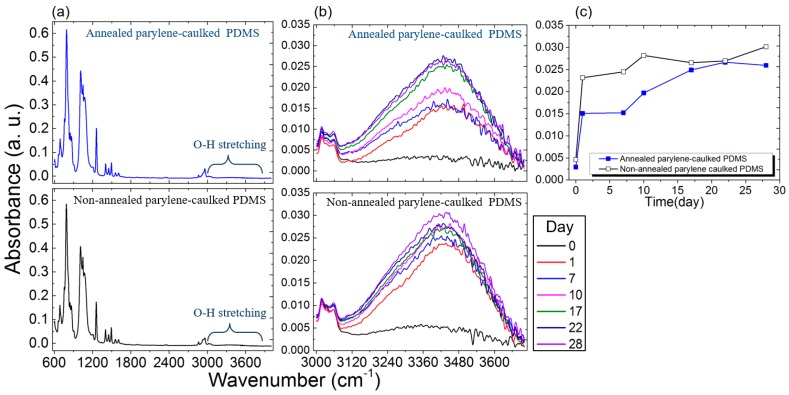
(**a**) Initial FTIR spectrum as soon as the electrodes were immersed in PBS solution; (**b**) FTIR spectrum from 3000 cm^−1^ to 3700 cm^−1^ to show the water amount absorbed in substrates of annealed pc-PDMS and non-annealed pc-PDMS for comparison; and (**c**) temporal changes in IR spectrum at 3450 cm^−1^ to show the water amount absorbed in substrates of annealed and non-annealed pc-PDMS.

In a previous study [15], water absorption into pc-PDMS was reported to be lower than that into native PDMS. Hence, in the present study, water absorption into annealed and non-annealed pc-PDMS was measured and compared. Three samples each of annealed and non-annealed pc-PDMS were used, and only one representative sample from each group is presented in Figure 3 as all the used samples showed the same trend. The initial spectra of annealed and non-annealed pc-PDMS are shown in Figure 3a, where no differences were detected. Figure 3b shows the IR spectrum from 3000 cm^−1^ to 3700 cm^−1^ to show the water amount absorbed in substrates at different time points. Water molecules consist of H and O, which results in the appearance of O-H vibrations. The types of vibrations were symmetric and asymmetric stretching, as observed at ~3450 cm^−1^ and ~3756 cm^−1^ [23]. Initially, the increase of the water amount in annealed pc-PDMS was less than that in non-annealed pc-PDMS but it seemed to converge to similar saturated values after a month. Figure 3c shows the temporal changes in the water amount absorbed in the substrates at 3450 cm^−1^. The amount of water absorption in non-annealed pc-PDMS increased dramatically only at the initial stage while that in annealed pc-PDMS increased steadily at a slower rate for a longer period. Then, the amount of water absorbed in annealed and non-annealed pc-PDMS became similar after 17 days. It was speculated that due to the scalable depth of the FTIR scan, 1~2 μm, which was smaller than the thickness of the specimens, 150 μm, the amount of water absorption in the two substrates was seemingly saturated within the scan depth. Based on these results, it was concluded that annealed pc-PDMS showed lower water absorption than non-annealed pc-PDMS.

### 3.3. Impedance Characterization

**Figure 4:**
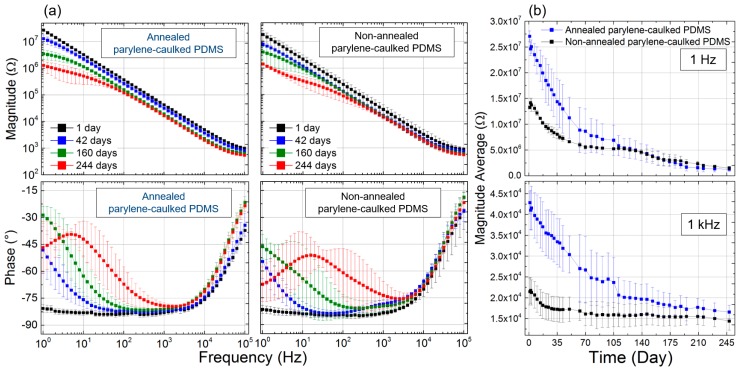
(**a**) Electrochemical impedance spectroscopy of the electrodes based on annealed and non-annealed parylene-caulked PDMS at day 1 to 244; and (**b**) temporal changes in impedance magnitude of the electrodes at 1 Hz and 1 kHz.

The dimensions of the fabricated electrode arrays were 40 mm in length and 2.5 mm in width, 850 µm × 850 µm for the electrical contact pads, 350 µm × 350 µm for the electrode sites, and 50-µm-wide metal traces. Figure 4 shows the impedance of the electrodes based on annealed and non-annealted pc-PDMS, for about eight months. The initial impedance magnitudes of the electrodes based on annealed and non-annealed pc-PDMS were 27.08 ± 1.04 MΩ and 13.27 ± 0.30 MΩ at 1 Hz, and 428.86 ± 2.58 kΩ and 214.99 ± 3.25 kΩ at 1 kHz, respectively, which indicates that annealing of the electrodes doubled the impedance. The reason for the higher impedance magnitude of annealed pc-PDMS is that the annealing process makes the electrode surface smoother [8,36]. While soaking the electrodes in PBS solution for 248 days, the impedance of the electrodes changed steadily. Firstly, the impedance phase of the electrodes based on non-annealed pc-PDMS shifted more than that of the electrodes based on annealed pc-PDMS. The impedance magnitudes of the electrodes based on annealed and non-annealed pc-PDMS at day 248 were 1.30 ± 0.50 MΩ and1.50 ± 0.25 MΩ at 1 Hz, and 164.89 ± 1.62 kΩ and 144.24 ± 2.25 kΩ at 1 kHz, respectively.

Until about 105 days, the impedance of the electrodes based on annealed pc-PDMS was higher than that of the electrodes based on non-annealed pc-PDMS. In addition, the electrode impedance based on both substrates converged to similar values after 200 days. The measured impedance magnitudes were comparable with those of other surface-type electrodes [37,38,39,40]. Annealing turned out to contribute to the increase in the electrode impedance without a reduction of the electrode areas. In some cases, a relatively high impedance of electrodes could be advantageous in recoding bio-signals with high noise levels, such as ECG or EEG signals, which are typically measured with poor contact between the target recording site and the electrode because of the persistent movements of internal organs such as the heart or muscle [41]. In term of the impedance phase, although the error bars of the phase were large, the phase of the non-annealed pc-PDMS impedance was certainly more shifted toward a high frequency than that of the annealed pc-PDMS. The reason for the phase shifts is that water diffuses into the polymer. Hence, polymeric substrates may swell and the dielectric constant of the substrates increases [42,43]. Therefore, based on the measured results of impedance phase and FTIR, we concluded that the water absorption of annealed pc-PDMS was lower than that of non-annealed pc-PDMS. As all dimensions were the same for the electrodes based on both substrates, it is reasonable that the impedance magnitude of each electrode converged to a simliar value after complete soaking. Thus, annealing effects on the the electrical property of the electrodes seemed to eventually disappear after eight months.

## 4. Conclusions

In this study, we investigated the influence of annealing pc-PDMS as a substrate of implantable electrodes. The annealing of pc-PDMS provided better mechanical strength compared to non-treated pc-PDMS, which indicates the electrodes based on annealed pc-PDMS may be more resistant to an in vivo environment where repeated relative movements persist. The water absorption of annealed pc-PDMS was lower than that of non-annealed pc-PDMS. Based on the impedance measurements, the impedance magnitude of annealed pc-PDMS was twice higher than that of pc-PDMS initially and was maintained higher for a certain period of time. After eight months of soaking in PBS solution, however, the impedance magnitude of the electrodes based on annealed and non-annealed pc-PDMS converged to similar values, which indicates that the annealing effects disapear after a couple of months in physiological environments. Thus, it is concluded that the annealing effects could be properly used in certain situations that require good mechanical strength of substrates or increased electrode impedance without reducing the electrode size. However, the annealing effect on the electrochemical properties of the electrodes did not last longer than a couple of months in physiological conditions. Such long-term annealing effects of pc-PDMS were investigated in the present study for the first time. It is believed that these results would be a good reference in biomedical engineering research.
